# Mini-clusters of postadenovirus VITT

**DOI:** 10.1016/j.rpth.2024.102641

**Published:** 2024-11-26

**Authors:** Michele P. Lambert, Theodore E. Warkentin

**Affiliations:** 1 Division of Hematology, Children’s Hospital of Philadelphia, Philadelphia, Pennsylvania, USA; 2 Department of Pediatrics, Perelman School of Medicine at the University of Pennsylvania, Philadelphia, Pennsylvania, USA; 3 Department of Pathology and Molecular Medicine, McMaster University, Hamilton, Ontario, Canada

Thrombotic events in pediatric patients are uncommon, with the risk of thrombosis in otherwise healthy children generally considered to be rare: 0.07 to 0.15 per 10,000 children [1,2], especially cerebral venous sinus thrombosis (CVST), with an estimated incidence of 0.67 cases per 100,000 children per year [3]. Most of these various types of thrombosis occur in neonates, adolescents, and hospitalized children, so their occurrence in school-age children who are otherwise well is exceedingly rare.

Certain specific risk factors have been identified for children that predispose to thrombosis, including antiphospholipid antibody syndrome, underlying malignancy, or infection. In the years since the initial description of COVID-19-associated thrombosis, it has been recognized that even pediatric patients demonstrate increased risk in this setting [4]. However, the pathophysiology of thrombosis with these predispositions is incompletely understood.

Platelet factor 4 (PF4)-related thrombotic/antibody disease is particularly rare in pediatrics; while immune heparin-induced thrombocytopenia is generally described in adult patients, it can occur in younger patients [5]. The spectrum of platelet-activating anti-PF4 disorders expanded in 2021 with the recognition of VITT, a platelet-activating, prothrombotic disorder caused by anti-PF4 antibodies that are rarely formed following COVID-19 vaccination with ChAdOx1 nCoV-19 [[6], [7], [8]] or Ad26.COV2.S [9], both engineered as adenoviral vector vaccines. More recently, a VITT-like disorder that occurs without proximate vaccination or heparin exposure but rather in the setting of recent infection, especially with adenovirus infection, has been described [[10], [11], [12], [13]]. Postviral VITT-like disorders appear to be rare and present acutely, with thrombosis in unusual sites, such as CVST or splanchnic vasculature, but also sometimes deep vein thrombosis (DVT) or pulmonary embolism, with concomitant thrombocytopenia and elevated D-dimer. Recent literature describes these atypical presentations of anti-PF4 disorders primarily in adult patients [13], although adenoviral-associated thrombocytopenia and thrombosis have been reported in both pediatric and adult patients [[10], [11], [12], [13], [14]].

In this issue, Dimopoulou et al. [15] describe a cluster of 3 pediatric patients observed in a 3-month period at a single institution, presenting with features that could be consistent with VITT-like syndrome. This geographic and temporal cluster contrasts with the previous pediatric case reports describing individual patients. All 3 patients were young (4-8 years old), previously healthy, and presented with acute symptomatic CVST with headache/altered mental status and vomiting/nausea. Laboratory features included thrombocytopenia, elevated D-dimer, and relatively decreased fibrinogen. Two patients had respiratory viral testing demonstrating adenoviral infection, and both patients tested for anti-PF4/heparin antibodies by commercially available lateral-flow immunoassay (LFI) showed positive results. Two patients were identified to be heterozygous for factor V Leiden. Treatment included anticoagulation and immunomodulation with high-dose intravenous immunoglobulin (IVIG) and 2 received corticosteroids.

This experience calls to mind a cluster of 3 pediatric patients one of us (M.P.L.) experienced over a 7-month period of time (July 2015 to February 2016) in a geographically localized area (greater Philadelphia; permission to report these cases has been provided by the patients’ parents). The children presented with severe spontaneous thrombosis that progressed with standard anticoagulation and required immunomodulation and alternative anticoagulation to halt progression (Table). At presentation, VITT had not yet been imagined, and the practice of using high-dose IVIG to block FcγIIa receptor-mediated platelet/cellular activation was not yet mainstream. These cases were managed as a thrombotic storm, an entity described by Ortel et al. [16].

**Table:** Clinical and laboratory features of a cluster of 3 pediatric patients with postviral thrombosis and thrombocytopenia and positive platelet factor 4/polyanion (polyvinyl sulfonate) enzyme-linked immunosorbent assay.

Clinical & laboratory data	Case 1	Case 2	Case 3
Age (y)/sex	2.75/F	5/M	4/F
Medical history	No	No	No
Initial symptoms	Cough, URI, vomiting, diarrhea, and headache	URI and swelling of LE	URI, fever, and AOM 2 wk before LE edema developed
Platelets on admission	**48 × 10**^**9**^**/L**	435 × 10^9^/L	**102 × 10**^**9**^**/L**
Platelet count nadir	**18 × 10**^**9**^**/L**	**87 × 10**^**9**^**/L**	**38 × 10**^**9**^**/L**
CRP, mg/dL	0.7	**6.5**	**5.0**
Fibrinogen, mg/dL	**134**	309	**144**
D-dimer, μg/mL	**>20** (0.27-0.6 μg/mL)	**>5** (0.1-0.6 μg/mL)	**>20** (0.27-0.6 μg/mL)
Respiratory viral testing	Adenovirus	Parainfluenza	Not done
Thrombophilia (inherited)	Heterozygous prothrombin gene mutation (G20210A0)	Heterozygous factor V Leiden	None
Anticardiolipin IgM/IgG, U/mL	<9/<9	Not done	<9/<9
β_2_ Glycoprotein I IgM/IgG, U/mL	<9/<9	Not done	<9/<9
DRVVT confirm (≤1.2)	1.1	1.2	**1.4**
Initial aPTT, s	39.5 (mildly prolonged)	29.7 (normal)	31.3 (normal)
PF4/PVS ELISA (IgG/A/M), commercial, units (0-27)	**230**	**40**	**63**
Celiac panel	Negative	Negative	Negative
Bone marrow aspirate/biopsy	Normal	Normal	Normal
Testing for paroxysmal nocturnal hemoglobinuria	Negative	Negative	Negative
Thromboses	CVST, LLE common femoral and superficial femoral DVT, R atrium	B/L LE DVT, PE, R subclavian vein, IVC, R atrium	L ext iliac, common femoral DVT, R ext iliac, common femoral DVT, IVC, L UE DVT, R atrium

Abnormal laboratory results are shown in **bold text**. D-dimer reference ranges are shown in parentheses.

AOM, acute otitis media; aPTT, activated partial thromboplastin time; B/L, bilateral; CRP, C-reactive protein; CVST, cerebral venous sinus thrombosis; DRVVT, dilute Russell’s viper venom time; DVT, deep vein thrombosis; ext, external; Ig, immunoglobulin; IVC, inferior vena cava; LLE, left lower extremity; LE, lower extremity; L UE, left upper extremity; PE, pulmonary embolism; PF4/PVS ELISA (IgG/A/M), platelet factor 4/polyvinyl sulfonate enzyme-linked immunosorbent assay, poly-specific (ie, detects IgG, IgA, and IgM class antibodies); R, right; URI, upper respiratory infection.

Case 1: a previously healthy 2-year-old girl presented with a 1-week history of diarrhea, headache, vomiting, and lethargy. She was irritable but had normal vital signs and a physical examination. Hemoglobin and white blood count were normal, but platelet count was 48 × 10^9^/L, dropping to 18 × 10^9^/L (nadir) the next day. The peripheral smear was only notable for thrombocytopenia. A diagnosis of immune thrombocytopenia was considered, and IVIG 1 g/kg was given. One day later, she had a worsening headache, lethargy, and persistent emesis. A brain computed tomography scan was concerning for CVST (superior sagittal sinus thrombosis); magnetic resonance venography confirmed extensive thrombosis of the superior sagittal and proximal right transverse sinuses. Additional workup showed normal prothrombin time, mildly prolonged activated partial thromboplastin time (39.5 seconds), mild hypofibrinogenemia (134 mg/dL), and elevated D-dimer (>20 μg/mL). Bone marrow biopsy was negative for malignancy. Extensive thrombophilia workup revealed only heterozygosity for the prothrombin gene mutation. She was transfused platelets and fresh frozen plasma prior to starting enoxaparin. An attempt at thrombolysis using a tissue-plasminogen activator (TPA) was initially successful, but there was immediate rethrombosis with the extension of the thrombus on magnetic resonance venography. Thrombocytopenia recurred on heparin, and she had a positive PF4/polyanion enzyme-linked immunosorbent assay (ELISA; Immucor) with a negative serotonin-release assay (SRA). Viral testing was positive for adenovirus. She also developed peripherally inserted central catheter-associated DVT and an intracardiac thrombus. A diagnosis of “thrombotic storm” was made, and she was treated with rituximab, therapeutic plasma exchange (TPE), and corticosteroids and was switched from heparin to bivalirudin. She improved significantly and was discharged on warfarin with corticosteroid weaning. She has remained thrombosis-free on warfarin for 8 years.

Case 2: a previously healthy 5-year-old boy presented with unprovoked symptomatic right femoral DVT in the setting of a recent parainfluenza virus infection. He was treated initially with enoxaparin and discharged. Two days later, he represented to the emergency department with acute worsening of lower extremity pain bilaterally and dyspnea and was found to have bilateral iliofemoral DVT with subsegmental pulmonary embolism. He was initially treated with enoxaparin and an attempt at TPA thrombolysis but progressed and developed subclavian vein DVT as well as noncontiguous right atrial thrombosis. He was transitioned to bivalirudin, started on corticosteroids, and underwent TPE. The initial hemoglobin was 10.5 g/dL with a platelet count of 435 × 10^9^/L. Thrombosis progression was accompanied by subsequent platelet count decrease from 270 to 87 × 10^9^/L (nadir), at which time transition to bivalirudin occurred. Thrombophilia evaluation demonstrated factor V Leiden heterozygosity and positive PF4/polyanion ELISA but negative SRA. No evidence of malignancy, inflammatory bowel disease, or rheumatologic disorder was found. He was transitioned to warfarin with corticosteroid weaning. He has remained thrombosis-free on warfarin for 8 years.

Case 3: a previously healthy 4-year-old girl presented 2 weeks after acute febrile upper respiratory illness with acute otitis media with unprovoked DVT of the left external iliac and common femoral veins. Initial hemoglobin was normal (12.9 g/dL) with mildly decreased platelet count (102 × 10^9^/L). She was started on heparin infusion, and because of the age and degree of limb symptoms, she received TPA thrombolysis, during which time thrombosis extended to the right iliofemoral vein despite a decrease in fibrinogen and increase in heparin dose from 20 to 27 units/kg/h. Thrombolysis was discontinued, with a transition to bivalirudin and the addition of corticosteroids. The platelet count declined to 38 × 10^9^/L (nadir). Viral testing was never performed. She was noted to have discontinuous right atrial thrombosis plus thrombosis of the inferior vena cava and left upper extremity thrombosis. She was started on TPE for 5 days and then treated with rituximab. PF4/polyanion ELISA was positive with negative SRA, and extensive thrombophilia evaluation was negative, as was evaluation for other causes for thrombosis (malignancy, inflammatory bowel disease, and rheumatologic). She was transitioned to warfarin, and corticosteroids were weaned. She has remained thrombosis-free on warfarin for 8 years.

These cases share similarities with the described cluster in the report by Dimopoulou et al. [15]. First, and most notably, the Greek group described 3 children with thrombocytopenia, elevated D-dimer, and unusual thrombosis (CVST) in the setting of recent respiratory viral infection (2 with adenovirus); the Philadelphia patients had unusual multisite thromboses (including CVST in 1 patient) following viral infection (1 with adenovirus). In both groups, 2 of 3 children had mild thrombophilia identified but no other inciting reason for such significant thromboses. Positive anti-PF4 serology was a feature of both reports (Athens, LFI and Philadelphia, ELISA), although, for the Greek patients, the finding that the PF4/heparin LFI was positive is perhaps unexpected, as this test usually yields negative results with VITT and thus presumably with VITT-like disorders [17]. All 3 Philadelphia patients tested with a PF4/polyanion ELISA demonstrated positive assays (Table). Clinicians should be aware of potential pitfalls in the diagnostic sensitivity of certain PF4-dependent immunoassays for the detection of postviral VITT-like antibodies [[10], [11], [12], [13]].

An important observation suggested from the Athens and Philadelphia pediatric group experiences is that because of the epidemic nature of a viral illness, even when an ultrarare complication such as postviral VITT-like disorder is plausible, there may arise apparent clusters of patients presenting with aggressive thromboses, thrombocytopenia, and coagulopathy (especially, decreased fibrinogen and greatly elevated D-dimer) in a postviral setting and without other risk factors or only with mild thrombophilia. This concept, of course, is applicable to the discovery of VITT itself: when tens of millions of otherwise healthy adults were vaccinated over several weeks in various geographic distributions with the adenoviral vector vaccines SARS-nCoV-19 and, subsequently, Ad26.COV2.S [18], clusters of VITT ensued despite its ultrarare nature; the medical/scientific community was alerted, and the anti-PF4 immunopathogenesis of VITT was quickly elucidated. The Athens and Philadelphia experiences will no doubt be replicated in other medical communities in the future.
